# TULAA: A Minimally Invasive Appendicectomy Technique for the Paediatric Patient

**DOI:** 10.1155/2016/6132741

**Published:** 2016-12-18

**Authors:** Giordano Perin, Maria Grazia Scarpa

**Affiliations:** Paediatric Surgery and Urology, Institute of Maternal and Child Health, IRCCS Burlo Garofolo, Trieste, Italy

## Abstract

TULAA or Transumbilical Laparoscopic Assisted Appendicectomy is a minimally invasive technique described by Pelosi in 1992 for the removal of the inflamed appendix. Its main advantage is the possibility of exploring the peritoneal cavity and performing a simple and safe extracorporeal appendicectomy. Since its first description, different authors reported their experience with such technique. The aim of this review is to summarise the surgical outcomes currently reported in the literature for this minimally invasive surgical approach and compare it with standard open and laparoscopic appendicectomy.

## 1. Introduction

The first literature report of a technique for the removal of the inflamed appendix dates back to Amyand in 1735 [1]: the surgeon removed the inflamed appendix of a young child that swallowed a pin. The appendix was found in an inguinal hernia sac and was perforated by the swallowed pin. In 1889, once made professor of Surgery [2], Charles McBurney highlighted the importance of early diagnosis and early surgical treatment of such a condition. Since then, many different techniques have been described. The first description of a laparoscopic appendicectomy in an adult population dates back to Semm in 1983 [3]. Such a technique became widely popular in the following years and is now considered a standard treatment for acute appendicitis. In 1992, Pelosi described an innovative surgical approach [4]: a Transumbilical Laparoscopic Assisted Appendicectomy (TULAA). This technique requires the use of an initial laparoscopic approach with pneumoperitoneum via a single umbilical port, the externalisation of the inflamed appendix via that port, and the removal of the appendix itself via a standard extracorporeal appendicectomy. Since then, this technique became quite popular especially in some European countries and has been shown to be particularly effective in the paediatric population. The main advantage of this approach is combining the possibility of exploring the peritoneal cavity and performing a simple and safe extracorporeal appendicectomy with a single small umbilical incision. The aim of this review is to summarise the results reported in the literature especially concerning the surgical outcomes (surgical time, hospital stay, and complications) and compare such results with what is reported for open and laparoscopic appendicectomy.

## 2. Materials and Methods

We searched PubMed and Google Scholar using the keywords “Transumbilical Laparoscopic assisted Appendicectomy”, “Transumbilical Laparoscopic assisted Appendectomy”, “Single Port Appendicectomy”, and “Single Port Appendectomy”. After analysing the available abstracts, we selected only the papers reporting results related to a laparoscopic assisted extracorporeal appendicectomy technique with umbilical access (initial laparoscopic approach via a single umbilical port and pneumoperitoneum, minimal dissection of the identified appendix with or without the insertion of a second port, externalisation of the inflamed appendix via the umbilical port and deflation of the pneumoperitoneum, and removal of the appendix itself via a standard extracorporeal technique and closure). A total of 24 papers, published between 1998 and 2015, were finally selected and reviewed. All the papers analysed were in English language. Seven were available in the form of abstracts; the remaining 17 were available in full text. Only 2 papers comparing Transumbilical Laparoscopic Assisted Appendicectomy with a standard open appendicectomy were found. Only 3 papers comparing Transumbilical Laparoscopic Assisted Appendicectomy with a standard laparoscopic appendicectomy were retrieved. Only 2 papers comparing outcomes between Transumbilical Laparoscopic Assisted Extracorporeal Appendicectomy, open appendicectomy, and laparoscopic appendicectomy were found.

## 3. Results

24 studies were found and analysed. Four involved a mixed population of adults and children; 20 described only paediatric cases. Inclusion and exclusion criteria were extremely variable and not always available (see Table 1). The specific technique in terms of equipment used (laparoscope, type of port, and number of ports used) is variable across the analysed studies; a detailed report can be found in Table 1. The number of patients enrolled ranged from 11 to 500. Only retrospective analyses were available; no prospective cohort studies were found.

The overall operating time reported in the literature ranges from 10 to 196 minutes. The use of descriptive statistic methods varies across the analysed literature; some authors prefer to report their outcome as mean operating time, and some authors prefer to use a median value. Overall, the mean/median operating time reported ranges from 15 to 58.6 minutes (see Table 2 for details). The overall hospital stay reported ranges from 1 to 89 days. Again, the use of descriptive statistic methods varies across the analysed papers; the mean/median hospital stay reported ranges from 2 to 7.9 days.

The need to use one or more additional ports to complete the appendicectomy ranges from 0 to 26.9%. The rate of conversion to open appendicectomy ranges from 0 to 15%.

The surgical wound infection rate ranges from 0 to 13.7% and the rate of intra-abdominal abscess ranges from 0 to 3.8%.

A full summary of the analysed literature is available in Table 1 (selection criteria, technique, and laparoscope used) and Table 2 (surgical time, hospital stay, and complication rate).

Only two studies comparing Transumbilical Laparoscopic Assisted Appendicectomy with a standard open technique were identified. Pappalepore and colleagues in 2002 [8] retrospectively analysed the records of 65 children undergoing a standard extracorporeal appendectomy and 58 patients undergoing a two-port Transumbilical Laparoscopic Assisted Extracorporeal Appendicectomy: 1 surgical wound infection was recorded in the first group, and no complications were recorded in the second group. Koizumi and colleagues in 2015 [22] retrospectively compared 64 patients undergoing open appendicectomy with 62 patients undergoing a single port Transumbilical Laparoscopic Assisted Extracorporeal Appendicectomy. 8/64 patients developed a complication in the first group (5 surgical wound infections, 1 intra-abdominal abscess, and 2 cases of ileus); 12/62 patients developed a complication in the second group (9 surgical wound infections, 1 abscess, and 2 cases of ileus).

Only three studies comparing Transumbilical Laparoscopic Assisted Appendicectomy with a standard laparoscopic approach were found. Visnjic [13] compared their outcomes with three different appendicectomy techniques: laparoscopic appendicectomy with staplers (34), laparoscopic appendicectomy with loops (9), and Transumbilical Laparoscopic Assisted Appendicectomy (29). 43 patients underwent one of the two laparoscopic technique appendicectomies and 3 developed a complication (2 wound infections and 1 intra-abdominal abscess). 29 patients underwent a Transumbilical Laparoscopic Assisted Appendicectomy and 4 developed a wound infection. Ostlie [19] in 2011 compared two groups of 180 patients undergoing a standard laparoscopic appendicectomy and Transumbilical Laparoscopic Assisted Extracorporeal Appendicectomy. 1.7% of the patients in the first group and 3.3% of the patients in the second group developed a wound infection. Finally, Kulaylat and colleagues [26] compared 132 patients that underwent Transumbilical Laparoscopic Assisted Appendicectomy with 240 patients treated with a standard laparoscopic multiport appendicectomy. Median operating time was shorter for Transumbilical Laparoscopic Assisted Appendicectomy (1 h versus 0.6 h, *p* < 0.0001). Hospital stay was comparable in the two groups. Fourteen out of 240 (5.8%) patients in the laparoscopic appendicectomy group and 9/132 (6.8%) patients in the Transumbilical Laparoscopic Assisted Appendicectomy group required a readmission. In each group, one case of surgical wound infection was recorded (resp., 0.42% in the laparoscopic appendicectomy group and 0.75% in the TULAA group). We recorded nine (3.75%) cases of intra-abdominal abscess in the laparoscopic appendicectomy group and 5 (3.8%) in the Transumbilical Laparoscopic Assisted Appendicectomy group.

Only two studies attempted to compare open appendicectomy, laparoscopic appendicectomy, and Transumbilical Laparoscopic Assisted Extracorporeal Appendicectomy. Bergholz and colleagues [24] compared 20 patients undergoing Transumbilical Laparoscopic Assisted Appendicectomy with 20 matched patients treated with laparoscopic appendicectomy and open appendicectomy, respectively. No significant difference between the three groups was found except for a slight increase in the analgesic requirements for the Transumbilical Laparoscopic Assisted Appendicectomy group. Scirè [27] and colleagues compared 46 patients treated with laparoscopic appendicectomy, 62 treated with Transumbilical Laparoscopic Assisted Appendicectomy, and 88 treated with open appendicectomy. They found an increase in wound infection rate and a reduction in length of hospital stay with Transumbilical Laparoscopic Assisted Extracorporeal Appendicectomy.

## 4. Discussion

The overall quality of the literature published so far is significantly limited: no prospective study analysing the outcomes of Transumbilical Laparoscopic Assisted Appendicectomy has been published. No randomised trial comparing the outcomes of standard techniques with Transumbilical Laparoscopic Assisted Appendicectomy has been identified. It is important to highlight that a significant number of the published reports (12/24) enrolled a limited number of patients, less than 100. If we exclude those small studies, the range of surgical complications rate changes significantly (see Table 3 for details). Overall results seem to be comparable with what is reported in the literature for both a mixed population [29] (Table 3) and a paediatric population [30] (Table 4).

Inclusion and exclusion criteria as well as preoperative investigations vary significantly across the analysed studies. Valla and colleagues [6] exclude patients affected by peritonitis, with a palpable mass or abscess, but do not specify by which means such conditions are diagnosed. While peritonitis and palpable mass are both clinical diagnosis, a confirmatory ultrasound may be needed to assess the presence of an abscess. Pappalepore [8] and colleagues included only patients with uncomplicated appendicitis: again they do not specify what preoperative investigations were carried out to assess the presence or the absence of complications. Koontz and colleagues [11] excluded all patients with documented perforated appendix at computer tomography: those patients were initially treated with antibiotics and an interval Transumbilical Laparoscopic Assisted Appendicectomy was performed at 4–6 weeks. Guanà and colleagues excluded patients with evidence of perforated appendix: all patients included were evaluated on the basis of biochemical (full blood count, C reactive protein) and radiological (ultrasound of the abdomen) investigations. Sesia and colleagues [15] adopted a similar approach. Saber [16] and colleagues used ultrasound or computed tomography to exclude patients with a perforated appendix or an abscess. Codrich and colleagues [21] used ultrasound to assess the presence of an appendiceal mass and schedule patients for an immediate appendicectomy or an interval/delayed appendicectomy. Koizumi and colleagues [22] excluded patients with evidence of peritonitis. Noviello and colleagues [28], finally, excluded patients affected by complicated appendicitis based on clinical and ultrasound findings: patients affected by complicated appendicitis were treated with open appendicectomy. The heterogeneity of the preoperative protocols adopted in the management of these patients might have influenced the reported surgical outcomes. In this context, it is important to highlight that only one study [21] explicitly enrolled all patients with acute appendicitis regardless of the initial clinical, radiological, or biochemical presentation.

The techniques described in the literature are extremely different between each other and in three out of 24 analysed studies details of the technique are not reported. The most commonly used technique reported in the literature requires the use of an operative laparoscope. We analysed 24 studies, ten of which involved the use of this kind of laparoscope, which carries the invaluable advantage of allowing the surgeon to visualise and perform dissection using a single port. Alternative described techniques involve the use of two trocars: the second trocar can be positioned suprapubically [8] or directly through the same umbilical incision [16, 26]. Ostlie [19] described the use of a second instrument inserted directly via an umbilical fascial incision, without the use of an additional port. Such a variety in the surgical technique could potentially affect the quality of the surgical outcome.

The operating time reported in the literature for Transumbilical Laparoscopic Assisted Appendicectomy is extremely variable. As mentioned, this range from 10 to 223 minutes and the mean or median operating time reported ranges from 15 to 58.6 minutes. If we exclude studies including less than 100 patients, the mean or median operating time does not change significantly (see Tables 3 and 4 for details). It is important to highlight, however, that the operating time is not significantly different from what is expected to be the operating time needed for a standard open or laparoscopic appendicectomy in a mixed population [29] or in a paediatric population [30]. These results may be biased by the lack of inclusion and exclusion criteria and a comparison to standard techniques may not be reliable.

The overall hospital stay reported in the literature for Transumbilical Laparoscopic Assisted Appendicectomy ranges from 1 to 89 days. Again, if we exclude the smaller studies (less than 100 patients enrolled), the picture does not change. It is important to highlight, however, that if we exclude the unfortunate case reported by Ohno and colleagues [20], the range drops to 1 to 22 days. The reported mean or median hospital stay ranges from 2 to 7.9 days. This is comparable with what we would expect for a standard or open laparoscopic appendicectomy in a mixed population [29] or in a paediatric population [30]. Again, the lack of clear inclusion and exclusion criteria makes the comparison difficult.

The reported need for one or more additional ports ranges from 0 to 26.9%. If we exclude the smallest reports (including less than 100 patients), this ranges from 0 to 18.6%. This is an extremely important factor to take into account: one-fifth of the cases required the use of an additional port. Unfortunately, the number of cases requiring the use of two additional ports (conversion to standard laparoscopic appendicectomy) is poorly documented in the literature.

The conversion rate reported ranges from 0 to 15%. If we exclude the smallest studies (including less than 100 patients), this does not change. These figures are not that different but still are slightly higher than the one normally reported in the literature for a laparoscopic appendicectomy in the adult and mixed population [29–32]. If we consider a paediatric population [30], however, the conversion rate is comparable.

The quality of the data related to complication rate is poor. The follow-up period is extremely variable in the published literature and often not reported (see Table 2).

Overall, we can say that wound infection rate ranges from 0 to 13.7%. If we exclude the smallest studies (less than 100 patients enrolled), this ranges from 0 to 6.3%. If we compare the data with what is reported for open appendicectomy in the adult and paediatric population, we realise that the figures are extremely similar and higher than what is reported for laparoscopic appendicectomy. The argument that performing an extracorporeal appendicectomy with the inflamed appendix going through the umbilical wound increases the infection rate compared to a laparoscopic approach seems to be true.

Organ space infection (intra-abdominal abscess) is reported in a rate variable from 0 to 3.8%. If we compare this data with what is known for a mixed adult and paediatric population [29], we realise that these figures are slightly higher. On the other hand, if we compare the data with what is reported in the literature for the paediatric population, there is basically no difference [30]. This could be due to multiple factors: first of all, the lack of inclusion and exclusion criteria, the fact that the population is mostly paediatric, and finally the fact that Transumbilical Laparoscopic Assisted Appendicectomy is a technique much less standardised compared with open and laparoscopic appendicectomy. Performing an extracorporeal appendicectomy with a purse string suture to bury the appendiceal stump seems not to reduce the risk of intra-abdominal collection.

Another very important point is related to costs: using one single port and simple instruments, the technique itself is cheaper than a standard laparoscopic appendicectomy as shown by different authors [11, 13, 18, 26]. Visnjic [13] compared their outcomes with three different appendicectomy techniques: laparoscopic appendicectomy with staplers (34), laparoscopic appendicectomy with loops (9), and Transumbilical Laparoscopic Assisted Appendicectomy (29). Considering only the cost of consumables used in the three techniques, they highlight how Transumbilical Laparoscopic Assisted Appendicectomy is cheaper as it involves the use of single multifilament absorbable suture instead of staples or endoloops. Kulaylat and colleagues [26] analysed their experience with 132 patients treated with Transumbilical Laparoscopic Assisted Appendicectomy and 240 patients treated with standard laparoscopic appendicectomy. They concluded that Transumbilical Laparoscopic Assisted Appendicectomy is comparable to laparoscopic appendicectomy in terms of surgical outcomes but is overall cheaper. They identified a statistically significant difference in favour of Transumbilical Laparoscopic Assisted Appendicectomy considering both admission and overall cost (including costs associated with readmissions for treatment of complications). It is difficult to identify what factors contributed in generating such difference, but it is overall important to highlight how both operative time and overall length of stay were shorter for patients treated with Transumbilical Laparoscopic Assisted Extracorporeal Appendicectomy. Another relevant factor to consider is that the number of patients treated with laparoscopic appendicectomy found to have a perforated appendix at operation was significantly higher, which could have contributed to increase the overall cost associated with this procedure. Stylianos and colleagues [18] reported their experience with 508 children treated with Transumbilical Laparoscopic Assisted Appendicectomy and compared their surgeon directed disposable supply costs with 17 other children's hospitals. Overall, their single port appendicectomy technique was recorded to be the cheapest. The main factor driving such difference was identified in the use of endomechanical devices which are clearly not required in Transumbilical Laparoscopic Assisted Extracorporeal Appendicectomy.

This technique seems to be particularly helpful in the paediatric population. This is especially true for few reasons. First of all, the value of a diagnostic laparoscopy is particularly significant in the paediatric population: it allows visualisation of the intra-abdominal content minimising the exposure to radiations, even when the diagnosis is in doubt. This is especially relevant if we consider that the resection of Meckel diverticulum can be performed via a single umbilical incision (Laparoscopic Assisted Resection of Meckel's Diverticulum or LATUM [33]). A second very important point is related to the mobilisation of the caecum: while on one side the relatively smaller size of the paediatric patient may represent a challenge for laparoscopic surgery, it makes it easier to externalise the appendix via the umbilical incision. A final relevant aspect to consider is related to the aesthetic impact: few authors highlighted how single port surgery has the potential of leaving a “scarless” abdomen [25, 26].

A further essential aspect to consider in this context is related to the learning curve. Again the quality of the literature available is poor: no specific learning curve study has been published so far and is difficult to identify a single surgical or clinical outcome to determine the shape of such curve. The main issue in this specific case is related to acquiring familiarity with the use of an operative laparoscope. As mentioned before, this seems to be the instrument most commonly used to perform a single port appendicectomy. Using an operative laparoscope requires the surgeon to to adapt to counterintuitive movements and tolerate a high degree of instrument conflict and frequent change in point of view. This could partly justify the high conversion rate and the need for use of further ports to perform the appendicectomy itself. Valla and colleagues [6] highlight how the number of cases that required the use of an additional port to perform the appendicectomy significantly declined following the first 100 procedures: from 7 out of 100 to 2 out of 94. Koontz and colleagues [11] highlight how the use of a procedure such as Transumbilical Laparoscopic Assisted Appendicectomy allows the surgeon to practice and maintain both laparoscopic and open surgery skills. Codrich and colleagues [21] highlight how 75% of the conversions recorded were performed by nonexpert members of the staff and 66% of the conversions to open surgery were performed in the first two years of the study. They highlight how the introduction of this procedure could help introduce less experienced surgeons to laparoscopy. Nicola [23] mentioned a specific number of procedures needed to overcome the learning curve: while for laparoscopic appendicectomy 15 procedures are needed, for Transumbilical Laparoscopic Appendicectomy 10 are deemed sufficient. Unfortunately, the author does not report a specific evidence to support such a statement. Nicola also highlight how in their experience most of the conversions to open technique or standard laparoscopic technique were performed in the first year of the study. If we compare the results reported in the literature for the learning curve for laparoscopic appendicectomy [34], we realise that the learning curve is generally comparable or even shorter. Kim and colleagues [34] report how to overcome the learning curve for laparoscopic appendicectomy; a surgical trainee would need to perform 30 procedures. Unfortunately, due to the fact that the data available in the literature is of poor quality, it is impossible to provide a reliable picture.

A final relevant aspect to consider in this context is the applicability of such technique to acute appendicitis complicated with localised abscess or mass. The approach to such condition is extremely different in the reported literature. Valla and colleagues [6], Noviello and colleagues [28], Sesia and colleagues [15], and Varshney and colleagues [12] decided not to consider Transumbilical Laparoscopic Assisted Extracorporeal Appendectomy a feasible technique in the presence of an abscess or appendiceal mass. On the other hand, Codrich and colleagues [21] and Gupta and colleagues [25] decided not to exclude such patients. Codrich and colleagues report their experience with 7 patients diagnosed with appendicular mass at admission: such patients were treated initially conservatively with antibiotics and all underwent a safe interval Transumbilical Laparoscopic Assisted Appendicectomy 8 weeks later. A similar experience is reported by Gupta and colleagues [25]: seven of the enrolled patients were identified as having an appendicular mass and were treated with antibiotics and interval Transumbilical Laparoscopic Assisted Appendicectomy 6 weeks later. According to the limited data available in the literature, Transumbilical Laparoscopic Assisted Appendicectomy should be considered as an alternative to open technique or laparoscopic technique for interval appendicectomy.

## 5. Conclusions

Transumbilical Laparoscopic Assisted Appendicectomy seems to be a safe and effective technique when compared with open and laparoscopic appendicectomy in a paediatric population. Wound infection rate seems to be higher with Transumbilical Laparoscopic Assisted Appendicectomy and open appendicectomy compared with laparoscopic appendicectomy. The main advantage of this approach is combining the possibility of exploring the peritoneal cavity and performing a simple and safe extracorporeal appendicectomy with a single umbilical incision. The overall quality of the published literature was found to be poor: the absence of prospective randomised trials makes the comparison with standard techniques difficult and potentially biased.

## Figures and Tables

**Table 1:** Summary of the analysed literature including population, inclusion/exclusion criteria, and trocars and scopes used.

Author	Population number of patients, age in years	Selection criteria	Trocar(s)	Scope
Esposito 1998 [5]	51 patients 4–16 (*μ* 7)	Not specified	Single trocar 10 mm	Operative scope

Valla et al. 1999 [6]	200 patients 5–18 (*μ* 9)	No peritonitis No abscess No palpable mass	Single trocar 11 mm	Operative scope

Martino et al. 2001 [7]	40 patients, paediatric, age not specified	Not specified	Single trocar 10 mm	Operative scope

Pappalepore et al. 2002 [8]	58 patients paediatric, age not specified	Uncomplicated	Two trocars 10 mm and 5 mm	Normal scope

D'Alessio et al. 2002 [9]	150 patients 2.5–17.4	Not specified	Single trocar	Not specified

Rispoli et al. 2002 [10]	65 patients mixed population, age not specified	Not specified	Single trocar 10 mm	Operative scope

Koontz et al. 2006 [11]	111 patients *μ* 11 ± 3.2	All children with preoperative diagnosis of appendicitis	Single trocar 10 mm	Operative scope

Varshney et al. 2007 [12]	11 patients 12–56 y.o. (*μ* 34)	Not specified	Single trocar 10 mm	Operative scope

Visnjic 2008 [13]	29 patients 5–17 (*μ* 9.5)	Not specified	Single trocar	Not specified

Guanà et al. 2010 [14]	231 patients *μ* 11.6 (3–18)	Exclusion of perforated appendicitis	Single trocar 11 mm	Operative scope

Sesia et al. 2010 [15]	262 *μ* 11.4 (1.1–15.9)	Exclusion of perforated appendix suspected on USS	Single trocar 12 mm with 5 mm working channel	Normal scope

Saber et al. 2010 [16]	26 patients M 33 (13–64)	Exclusion of perforated appendix or abscess at USS or CT	Two trocars (12 mm and 5 mm) through the same umbilical incision	Normal scope

Shekherdimian and DeUgarte 2011 [17]	21 patients paediatric, age not specified	Not specified	Single trocar 3 or 5 mm	Normal scope, grasper inserted through wound

Stylianos et al. 2011 [18]	398 patients paediatric, age not specified	Not specified	Single trocar 12 mm	Operative scope

Ostlie 2011 [19]	180 patients *μ* 11.5 ± 3.47	Not specified	Single trocar 12 mm plus fascial incision	Normal scope, instruments inserted through fascial incision

Ohno et al. 2012 [20]	500 patients M 10.2 (2–16)	Not specified	Single trocar 12 mm used with 2 instruments	Normal scope, grasper inserted via the same port

Codrich et al. 2013 [21]	203 patients *μ* 10 (3–17)	As per study protocol	Single trocar 11 mm	Operative scope

Koizumi et al. 2015 [22]	94 patients *μ* 41.1 (13–89)	Exclusion of patients with peritonitis	Single trocar, triport	Normal scope

Nicola 2014 [23]	120 patients *μ* 9.9 (6–14)	0–14 years old, uncomplicated appendicitis	Single trocar 10 mm	Operative scope

Bergholz et al. 2014 [24]	20 patients Not reported	Not reported	Not reported	Not reported

Gupta et al. 2014 [25]	58 patients *μ* 10.2 (3–16)	Only interval appendectomy	Single trocar 5 mm	Normal scope, second instrument inserted through facial incision

Kulaylat et al. 2014 [26]	132 patients *μ* 9.4 (±3.8)	Not specified	2 × 5 mm trocars inserted through two umbilical fascial incisions	30 degrees 5 mm scope

Scirè et al. 2014 [27]	62 patients Not specified	Not specified	Not specified	Not specified

Noviello et al. 2015 [28]	300 patients	Uncomplicated appendicitis	Not reported	Not reported

**Table 2:** Summary of the analysed literature including surgical time (in minutes), length of hospital stay (in days unless otherwise specified), and complications/need for additional ports.

Author	Population number of patients, age in years	Surgical time (minutes)	Hospital stay (days)	Complications/need for additional ports
Esposito 1998 [5]	51 patients 4–16 (*μ* 7)	M 25 (12–45)	M 2 (1–4)	None

Valla et al. 1999 [6]	200 patients 5–18 (*μ* 9)	M 15 (10–90)	M 2 (1–22)	16 (8%): insertion of another trocar 3 (1.5%): parietal complications 7 (3.5%): intra-abdominal complications

Martino et al. 2001 [7]	40 patients, paediatric, age not specified	M 50.9 (30–120)	Not specified	Not specified

Pappalepore et al. 2002 [8]	58 patients paediatric, age not specified	M 25	2–4	1: conversion to open (1.7%) 1: additional trocar (1.7%)

D'Alessio et al. 2002 [9]	150 patients 2.5–17.4	*μ* 35	*μ* 3.5	28: additional trocar (18.6%) 6: conversions to OA (4%)

Rispoli et al. 2002 [10]	65 patients mixed population, age not specified	M 25 (15–70)	M 2 (1–4)	5: additional trocar (7.7%) 5: conversions to OA (7.7%)

Koontz et al. 2006 [11]	111 patients *μ*11 ± 3.2	M 36 (9–140)	*μ* 1.8 (1–11)	2: additional trocar (1.8%) 2: conversions to OA (1.8%) 1: intra-abdominal abscess (0.9) 7: wound infections (6.3%)

Varshney et al.2007 [12]	11 patients 12–56 y.o. (*μ* 34)	M 20 (15–25)	M 1.5 (1-2)	None

Visnjic 2008 [13]	29 patients 5–17 (*μ* 9.5)	*μ* 39 (24–66)	M 3	4: wound infections (13.7%)

Guanà et al. 2010 [14]	231 patients *μ* 11.6 (3–18)	*μ* 38 (25–100)	*μ* 3.5 (3–7)	1: insertion of second trocar (0.43%) 1: insertion of two additional trocars (0.43%) 1: enlargement of initial incision 2: conversions to OA (8.6%)

Sesia et al. 2010 [15]	262 *μ* 11.4 (1.1–15.9)	Not specified	Not specified	3: intra-abdominal abscess (1.1%)

Saber et al. 2010 [16]	26 patients M 33 (13–64)	M 45.9 (30–80)	M 1.1 (0–2)	4: additional trocar (15.4%) 3: two additional trocars (11.5%)

Shekherdimian and DeUgarte 2011 [17]	21 patients paediatric, age not specified	*μ* 51 ± 15	*μ* 1.2 ± 0.8	3: conversions to OA (14.3%)

Stylianos et al. 2011 [18]	398 patients paediatric, age not specified	*μ* 24 (5–56)	Not reported	39: additional one or more trocars (9.8%)

Ostlie 2011 [19]	180 patients *μ* 11.5 ± 3.47	*μ* 35.2 ± 14.5	M < 1 day (22.7 ± 6.2 h)	3.3%: surgical site infection

Ohno et al. 2012 [20]	500 patients M 10.2 (2–16)	M 44.5 (10–169)	M 7.9 (1–89)	3: single additional trocar (0.6%) 11: two additional trocars (2.2%) 21: intraoperative complications (4.2%) 26: postoperative complications (5.2%)

Codrich et al. 2013 [21]	203 patients *μ* 10 (3–17)	M 52	Not reported	181: urgent operations 5: wound infections (2.5%) 5: abscess (2.5%)

Koizumi et al. 2015 [22]	94 patients *μ* 41.1 (13–89)	M 54 (23–223)	M 4.7 (1–33)	5: surgical site infection (5.3%) 1: abscess (1%) 2: ileus (2.1)

Nicola 2014 [23]	120 patients *μ* 9.9 (6–14)	*μ* 58.6 (14–135)	*μ* 3.7 (2–14)	11: additional trocar (9%) 8: conversion to LAP (6%) 5: surgical site infection (4%) 1: abscess (0.8%)

Bergholz et al. 2014 [24]	20 patients Not reported	Not reported	Not reported	Reported to be not significantly different from OA and LA group

Gupta et al. 2014 [25]	58 patients *μ* 10.2 (3–16)	M 52	*μ* 1.2 ± 0.8	3 cases converted to OA (5.2%)

Kulaylat et al. 2014 [26]	132 patients *μ* 9.4 (±3.8)	*μ* 36	M 4 (0.7–12)	1: surgical wound infection (0.75%) 5: intra-abdominal abscess (3.8%)

Scirè et al. 2014 [27]	62 patients Not specified	Not specified	Not specified	Similar complications in the three included groups (see text for details)

Noviello et al. 2015 [28]	300 patients	*μ* 42	Not reported	45: conversion to OA (15%) 3: conversion to LA (1%) 11: surgical site infection (3.7%)

*μ*: mean, M: median, and OA: open appendicectomy.

**Table 3:** Comparison of outcomes with College of Surgeons National Surgical Quality Improvement Program (NSQIP) database for 2005 to 2008, data published by Fleming et al. [29].

	Fleming et al. [29]		Range of mean/median or percentage value reported in the literature for TULAEA	
	Open appendicectomy	Laparoscopic appendicectomy	Including all studies	Including only studies with >100 patients
Operating time	49 minutes (M)	47 minutes (M)	15–58.6 minutes (M/*μ*)	15–58.6 minutes (M/*μ*)
Hospital stay	2.3 days	1.8 days	2–7.9 days (M/*μ*)	2–7.9 days (M/*μ*)
Conversion rate	N/A	1.9%	0–15%	0–15%
Surgical wound infection	5.2%	1.7%	0–13.7%	0–6.3%
Organ space infection	1.9%	1.8%	0–3.8%	0–3.8%

M: median, *μ*: mean, M/*μ*: range of median or mean values reported in the literature, and N/A: not applicable.

**Table 4:** Comparison of outcomes with meta-analysis data of open versus laparoscopic appendicectomy, data published by Aziz et al. [30].

	Aziz et al. [30]		Range of mean/median or percentage value reported in the literature for TULAEA	
	Open appendicectomy	Laparoscopic appendicectomy	Including all studies	Including only studies with >100 patients
Operating time	83 to 46 minutes (*μ*)	73 to 31 minutes (*μ*)	15–58.6 minutes (M/*μ*)	15–58.6 minutes (M/*μ*)
Hospital stay	2.4 to 6.5 days (*μ*)	1.70 to 7 days (*μ*)	2–7.9 days (M/*μ*)	2–7.9 days (M/*μ*)
Conversion rate	N/A	0 to 25.9%	0–15%	0–15%
Surgical wound infection	5%	1.5%	0–13.7%	0–6.3%
Organ space infection	3.4%	3.8%	0–3.8%	0–3.8%

M: median, *μ*: mean, M/*μ*: range of median or mean values reported in the literature, and N/A: not applicable.

## References

[1] Amyand C. (1735). Of an inguinal rupture, with a pin in the appendix coeci, incrusted with stone; and some observations on wounds in the guts. *Philosophical Transactions*.

[2] Yale S. H., Musana K. A. (2005). Charles Heber McBurney (1845–1913). *Clinical Medicine & Research*.

[3] Semm K. (1983). Endoscopic appendectomy. *Endoscopy*.

[4] Pelosi M. A. (1992). Laparoscopic appendectomy using a single umbilical puncture (minilaparoscopy). *The Journal of Reproductive Medicine*.

[5] Esposito C. (1998). One-trocar appendectomy in pediatric surgery. *Surgical Endoscopy*.

[6] Valla J.-S., Ordorica-Flores R. M., Steyaert H. (1999). Umbilical one-puncture laparoscopic-assisted appendectomy in children. *Surgical Endoscopy*.

[7] Martino A., Zamparelli M., Cobellis G., Mastroianni L., Amici G. (2001). One-trocar surgery: a less invasive videosurgical approach in childhood. *Journal of Pediatric Surgery*.

[8] Pappalepore N., Tursini S., Marino N., Lisi G., Lelli Chiesa P. (2002). Transumbilical laparoscopic-assisted appendectomy (TULAA): a safe and useful alternative for uncomplicated appendicitis. *European Journal of Pediatric Surgery*.

[9] D'Alessio A., Piro E., Tadini B., Beretta F. (2002). One-trocar transumbilical laparoscopic-assisted appendectomy in children: our experience. *European Journal of Pediatric Surgery*.

[10] Rispoli G., Armellino M. F., Esposito C. (2002). One-trocar appendectomy: sense and nonsense. *Surgical Endoscopy and Other Interventional Techniques*.

[11] Koontz C. S., Smith L. A., Burkholder H. C., Higdon K., Aderhold R., Carr M. (2006). Video-assisted transumbilical appendectomy in children. *Journal of Pediatric Surgery*.

[12] Varshney S., Sewkani A., Vyas S. (2007). Single-port transumbilical laparoscopic-assisted appendectomy. *Indian Journal of Gastroenterology*.

[13] Visnjic S. (2008). Transumbilical laparoscopically assisted appendectomy in children: high-tech low-budget surgery. *Surgical Endoscopy and Other Interventional Techniques*.

[14] Guanà R., Gesmundo R., Maiullari E. (2010). Treatment of acute appendicitis with one-port transumbilical laparoscopic-assisted appendectomy: a six-year, single-centre experience. *African Journal of Paediatric Surgery*.

[15] Sesia S. B., Haecker F.-M., Kubiak R., Mayr J. (2010). Laparoscopy-assisted single-port appendectomy in children: is the postoperative infectious complication rate different?. *Journal of Laparoendoscopic and Advanced Surgical Techniques*.

[16] Saber A. A., Elgamal M. H., El-Ghazaly T. H., Dewoolkar A. V., Akl A. (2010). Simple technique for single incision transumbilical laparoscopic appendectomy. *International Journal of Surgery*.

[17] Shekherdimian S., DeUgarte D. (2011). Transumbilical laparoscopic-assisted appendectomy: an extracorporeal single-incision alternative to conventional laparoscopic techniques. *The American Surgeon*.

[18] Stylianos S., Nichols L., Ventura N., Malvezzi L., Knight C., Burnweit C. (2011). The “all-in-one” appendectomy: quick, scarless, and less costly. *Journal of Pediatric Surgery*.

[19] Ostlie D. J. (2011). Single-site umbilical laparoscopic appendectomy. *Seminars in Pediatric Surgery*.

[20] Ohno Y., Morimura T., Hayashi S.-I. (2012). Transumbilical laparoscopically assisted appendectomy in children: the results of a single-port, single-channel procedure. *Surgical Endoscopy and Other Interventional Techniques*.

[21] Codrich D., Scarpa M. G., Lembo M. A. (2013). Transumbilical laparo-assisted appendectomy: a safe operation for the whole spectrum of appendicitis in children—a single-centre experience. *Minimally Invasive Surgery*.

[22] Koizumi N., Kobayashi H., Nakase Y., Takagi T., Fukumoto K. (2015). Efficacy of transumbilical laparoscopic-assisted appendectomy for appendicitis: a four-year experience at a single center. *Surgery Today*.

[23] Nicola Z. (2014). Transumbilical laparoscopic-assisted appendectomy in children: clinical and surgical outcomes. *World Journal of Gastrointestinal Endoscopy*.

[24] Bergholz R., Krebs T. F., Klein I., Wenke K., Reinshagen K. (2014). Transumbilical laparoscopic-assisted versus 3-port laparoscopic and open appendectomy: a case-control study in children. *Surgical Laparoscopy, Endoscopy and Percutaneous Techniques*.

[25] Gupta R. K., Gupta A., Kothari P., Kesan K. K., Dixit V., Kamble R. (2014). Transumbilical laparoscopic-assisted appendectomy: a safe and useful technique for interval appendectomy in pediatric population. *Journal of Minimally Invasive Surgical Sciences*.

[26] Kulaylat A. N., Podany A. B., Hollenbeak C. S., Santos M. C., Rocourt D. V. (2014). Transumbilical laparoscopic-assisted appendectomy is associated with lower costs compared to multiport laparoscopic appendectomy. *Journal of Pediatric Surgery*.

[27] Scirè G., Mariotto A., Peretti M. (2014). Laparoscopic versus open appendectomy in the management of acute appendicitis in children: a multicenter retrospective study. *Minerva Pediatrica*.

[28] Noviello C., Romano M., Martino A., Cobellis G. (2015). Transumbilical laparoscopic-assisted appendectomy in the treatment of acute uncomplicated appendicitis in children. *Gastroenterology Research and Practice*.

[29] Fleming F. J., Kim M. J., Messing S., Gunzler D., Salloum R., Monson J. R. (2010). Balancing the risk of postoperative surgical infections: a multivariate analysis of factors associated with laparoscopic appendectomy from the NSQIP database. *Annals of Surgery*.

[30] Aziz O., Athanasiou T., Tekkis P. P. (2006). Laparoscopic versus open appendectomy in children: a meta-analysis. *Annals of Surgery*.

[31] Brügger L., Rosella L., Candinas D., Güller U. (2011). Improving outcomes after laparoscopic appendectomy: a population-based, 12-year trend analysis of 7446 patients. *Annals of Surgery*.

[32] Sakpal S. V., Bindra S. S., Chamberlain R. S. (2012). Laparoscopic appendectomy conversion rates two decades later: an analysis of surgeon and patient-specific factors resulting in open conversion. *Journal of Surgical Research*.

[33] Prasad T. R. S., Chui C. H., Jacobsen A. S. (2006). Laparoscopic-assisted resection of Meckel's diverticulum in children. *Journal of the Society of Laparoendoscopic Surgeons*.

[34] Kim S. Y., Hong S. G., Roh H. R., Park S. B., Kim Y. H., Chae G. B. (2010). Learning curve for a laparoscopic appendectomy by a surgical trainee. *Journal of the Korean Society of Coloproctology*.

